# 4-[(2′-Cyano­biphenyl-4-yl)meth­yl]morpholin-4-ium hexa­fluoridophosphate

**DOI:** 10.1107/S160053681201358X

**Published:** 2012-04-13

**Authors:** Hua Yu Xue, Shi Juan Wang

**Affiliations:** a Department of Applied Chemistry, Nanjing College of Chemical Technology, Nanjing 210048, People’s Republic of China

## Abstract

In the cation of the title compound, C_18_H_19_N_2_O^+^·PF_6_^−^, the morpholine ring adopts the usual chair conformation and the dihedral angle between the benzene rings is 67.55 (11)°. The F atoms of the anion are disordered over two orientations with a refined occupancy ratio of 0.65 (2):0.35 (2). In the crystal, inter­molecular N—H⋯N hydrogen bonds link the cations into chains parallel to the *c* axis. The crystal packing is further enforced by inter­ionic C—H⋯F hydrogen bonds.

## Related literature

For the screening of mol­ecular salts with physicochemical properties, see: Tong & Whitesell (1998 ▶); Shanker (1994 ▶). For the structures of related salts, see: SiMa (2010 ▶); Li *et al.* (2011 ▶).
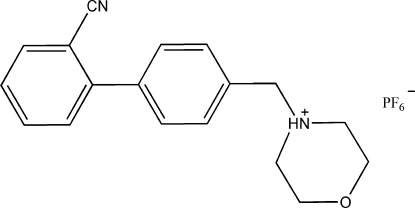


## Experimental

### 

#### Crystal data

C_18_H_19_N_2_O^+^·PF_6_^−^
*M* *_r_* = 424.32
Monoclinic, 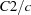

*a* = 24.912 (11) Å
*b* = 10.757 (5) Å
*c* = 14.925 (7) Å
β = 91.07 (3)°
*V* = 3999 (3) Å^3^
*Z* = 8
Mo *K*α radiation
μ = 0.20 mm^−1^
*T* = 293 K
0.20 × 0.20 × 0.20 mm

#### Data collection

Rigaku Mercury2 diffractometer
Absorption correction: multi-scan (*CrystalClear*; Rigaku, 2005 ▶) *T*_min_ = 0.813, *T*_max_ = 1.000
21155 measured reflections
4512 independent reflections
3216 reflections with *I* > 2σ(*I*)
*R*_int_ = 0.052

#### Refinement

*R*[*F* ^2^ > 2σ(*F* ^2^)] = 0.084
*wR*(*F* ^2^) = 0.218
*S* = 1.19
4512 reflections
308 parameters
H-atom parameters constrained
Δρ_max_ = 0.16 e Å^−3^
Δρ_min_ = −0.17 e Å^−3^

### 

Data collection: *CrystalClear* (Rigaku, 2005 ▶); cell refinement: *CrystalClear*; data reduction: *CrystalClear*; program(s) used to solve structure: *SHELXS97* (Sheldrick, 2008 ▶); program(s) used to refine structure: *SHELXL97* (Sheldrick, 2008 ▶); molecular graphics: *SHELXTL* (Sheldrick, 2008 ▶); software used to prepare material for publication: *SHELXTL*.

## Supplementary Material

Crystal structure: contains datablock(s) I, global. DOI: 10.1107/S160053681201358X/rz2727sup1.cif

Structure factors: contains datablock(s) I. DOI: 10.1107/S160053681201358X/rz2727Isup2.hkl

Supplementary material file. DOI: 10.1107/S160053681201358X/rz2727Isup3.cml

Additional supplementary materials:  crystallographic information; 3D view; checkCIF report

## Figures and Tables

**Table 1:** Hydrogen-bond geometry (Å, °)

*D*—H⋯*A*	*D*—H	H⋯*A*	*D*⋯*A*	*D*—H⋯*A*
N2—H2*A*⋯N1^i^	0.91	2.04	2.942 (4)	171
C10—H10*A*⋯F1^ii^	0.93	2.43	3.296 (9)	155
C14—H14*A*⋯F3	0.97	2.39	3.355 (12)	171
C15—H15*A*⋯F6	0.97	2.46	3.377 (8)	158
C15—H15*B*⋯F3^ii^	0.97	2.48	3.412 (10)	161
C15—H15*B*⋯F3′^ii^	0.97	2.54	3.51 (2)	172
C10—H10*A*⋯F1′^ii^	0.93	2.45	3.26 (2)	145
C14—H14*B*⋯F1′^ii^	0.97	2.38	3.097 (16)	130
C5—H5*A*⋯F2′^iii^	0.93	2.48	3.41 (2)	178
C17—H17*B*⋯F6′^iv^	0.97	2.47	3.29 (3)	143

Symmetry codes: (i) 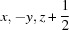; (ii) 
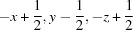
; (iii) 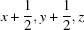; (iv) 
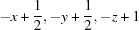
.

**Fig. 1.**
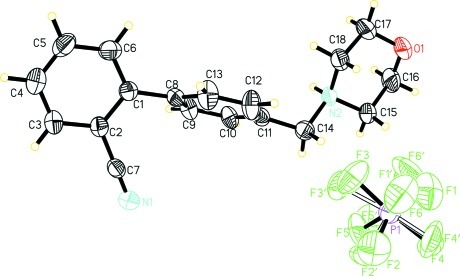
The molecular structure of the title compound, showing displacement ellipsoids drawn at the 30% probability level.

**Fig. 2.**
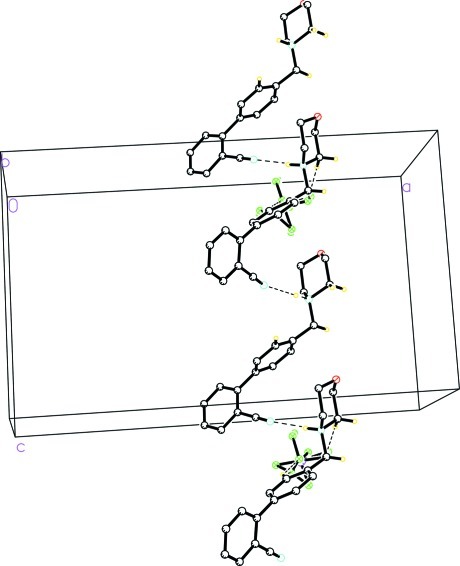
Partial crystal packing of the title compound showing a chain of cations interacting via intermolecular N—H···N hydrogen bonds (dashed lines).

## supplementary crystallographic information

### Comment

The title compound was prepared as part of our ongoing studies of hydrogen-bonding interactions in the crystal structure of protonated amines. The importance of molecular salts in pharmaceutical formulations is well known. For a given active ingredient, the isolation and selection of a salt with the appropriate physicochemical properties involves significant screening activity, as discussed at some length in the literature (Tong & Whitesell, 1998; Shanker, 1994). Here we report the synthesis and crystal structure of the title compound, 4-[(2'-cyanobiphenyl-4-yl)methyl]morpholin-4-ium hexafluorophosphate.

In the title compound (Fig. 1), bond distances and angles agree very well with those reported for a closely related nitrate (SiMa, 2010) and tetrafluoridoborate (Li *et al.*, 2011) derivatives. In the cation, the morpholine ring adopts the usual chair conformation, and the dihedral angle formed by the phenyl rings is 67.55 (11)°. The hexafluorophosphate anion displays a distorted octahedral geometry, the fluorine atoms being disordered over two orientations with site occupancies of 0.65 (2) and 0.35 (2) for the major and minor components of disorder, respectively. In the structure, the cations interact through intermolecular N–H···N hydrogen bonds (Table 1) to form chains parallel to the *c* axis (Fig. 2). Crystal packing is further consolidated by interionic C–H···O hydrogen bonds.

### Experimental

To a stirred solution of 4'-(morpholinomethyl)biphenyl-2-carbonitrile (5.56 g, 0.02 mol) in methanol (30 mL), hexafluorophosphoric acid (4.17 g, 0.02 mol) was added at the room temperature. The precipitate was filtered and washed with a small amount of ethanol 95%. Single crystals suitable for X-ray diffraction analysis were obtained from slow evaporation of a solution of the title compound in water at room temperature.

### Refinement

All H-atoms were positioned geometrically and refined using a riding model, with N—H = 0.91 Å, C—H = 0.93–0.96 Å and *U*_iso_(H) = 1.2 *U*_eq_(N, C). The fluorine atoms of the anion are disoreder over two orientations with a refined occupancy ratio of 0.65 (2):0.35 (2).

### Crystal data

**Table:**

C_18_H_19_N_2_O^+^·PF^6^^−^	*F*(000) = 1744
*M**_r_* = 424.32	*D*_x_ = 1.410 Mg m^−^^3^
Monoclinic, *C*2/*c*	Mo *K*α radiation, λ = 0.71073 Å
Hall symbol: -C 2yc	Cell parameters from 4512 reflections
*a* = 24.912 (11) Å	θ = 2.6–27.4°
*b* = 10.757 (5) Å	µ = 0.20 mm^−^^1^
*c* = 14.925 (7) Å	*T* = 293 K
β = 91.07 (3)°	Prism, colourless
*V* = 3999 (3) Å^3^	0.20 × 0.20 × 0.20 mm
*Z* = 8	

### Data collection

**Table:**

Rigaku Mercury2 diffractometer	4512 independent reflections
Radiation source: fine-focus sealed tube	3216 reflections with *I* > 2σ(*I*)
Graphite monochromator	*R*_int_ = 0.052
Detector resolution: 13.6612 pixels mm^-1^	θ_max_ = 27.4°, θ_min_ = 2.1°
CCD_Profile_fitting scans	*h* = −32→31
Absorption correction: multi-scan (*CrystalClear*; Rigaku, 2005)	*k* = −13→13
*T*_min_ = 0.813, *T*_max_ = 1.000	*l* = −19→19
21155 measured reflections	

### Refinement

**Table:**

Refinement on *F*^2^	Primary atom site location: structure-invariant direct methods
Least-squares matrix: full	Secondary atom site location: difference Fourier map
*R*[*F*^2^ > 2σ(*F*^2^)] = 0.084	Hydrogen site location: inferred from neighbouring sites
*wR*(*F*^2^) = 0.218	H-atom parameters constrained
*S* = 1.19	*w* = 1/[σ^2^(*F*_o_^2^) + (0.0935*P*)^2^ + 1.2795*P*] where *P* = (*F*_o_^2^ + 2*F*_c_^2^)/3
4512 reflections	(Δ/σ)_max_ < 0.001
308 parameters	Δρ_max_ = 0.16 e Å^−^^3^
0 restraints	Δρ_min_ = −0.17 e Å^−^^3^

### Special details

**Table:**

Geometry. All e.s.d.'s (except the e.s.d. in the dihedral angle between two l.s. planes) are estimated using the full covariance matrix. The cell e.s.d.'s are taken into account individually in the estimation of e.s.d.'s in distances, angles and torsion angles; correlations between e.s.d.'s in cell parameters are only used when they are defined by crystal symmetry. An approximate (isotropic) treatment of cell e.s.d.'s is used for estimating e.s.d.'s involving l.s. planes.
Refinement. Refinement of *F*^2^ against ALL reflections. The weighted *R*-factor *wR* and goodness of fit *S* are based on *F*^2^, conventional *R*-factors *R* are based on *F*, with *F* set to zero for negative *F*^2^. The threshold expression of *F*^2^ > σ(*F*^2^) is used only for calculating *R*-factors(gt) *etc*. and is not relevant to the choice of reflections for refinement. *R*-factors based on *F*^2^ are statistically about twice as large as those based on *F*, and *R*- factors based on ALL data will be even larger.

### Fractional atomic coordinates and isotropic or equivalent isotropic displacement parameters (Å^2^)

**Table:**

	*x*	*y*	*z*	*U*_iso_*/*U*_eq_	Occ. (<1)
N2	0.32167 (9)	0.0538 (2)	0.37953 (14)	0.0586 (6)	
H2A	0.3533	0.0143	0.3918	0.070*	
C11	0.36122 (12)	0.1175 (3)	0.23215 (17)	0.0587 (7)	
C2	0.49042 (11)	0.2243 (3)	−0.01594 (18)	0.0597 (7)	
C8	0.44407 (11)	0.2199 (3)	0.13211 (17)	0.0568 (7)	
N1	0.42775 (12)	0.0475 (3)	−0.07023 (19)	0.0804 (8)	
C1	0.48586 (12)	0.2708 (3)	0.07186 (18)	0.0598 (7)	
C3	0.52800 (13)	0.2724 (3)	−0.0749 (2)	0.0750 (9)	
H3A	0.5302	0.2411	−0.1328	0.090*	
C15	0.27767 (13)	−0.0213 (3)	0.4206 (2)	0.0729 (9)	
H15A	0.2431	0.0145	0.4041	0.088*	
H15B	0.2787	−0.1056	0.3977	0.088*	
C7	0.45498 (12)	0.1267 (3)	−0.04641 (19)	0.0624 (7)	
C10	0.40494 (13)	0.0482 (3)	0.21080 (19)	0.0679 (8)	
H10A	0.4072	−0.0341	0.2296	0.082*	
C9	0.44606 (13)	0.0986 (3)	0.16145 (19)	0.0685 (8)	
H9A	0.4756	0.0497	0.1478	0.082*	
C14	0.31444 (13)	0.0585 (3)	0.27939 (19)	0.0697 (8)	
H14A	0.2821	0.1050	0.2649	0.084*	
H14B	0.3095	−0.0255	0.2570	0.084*	
C12	0.35927 (15)	0.2401 (3)	0.2056 (3)	0.0877 (11)	
H12A	0.3301	0.2892	0.2210	0.105*	
O1	0.28375 (13)	0.0972 (4)	0.55657 (19)	0.1302 (13)	
C13	0.40029 (15)	0.2908 (3)	0.1561 (3)	0.0877 (11)	
H13A	0.3984	0.3738	0.1388	0.105*	
C6	0.52083 (15)	0.3653 (3)	0.0969 (2)	0.0810 (10)	
H6A	0.5191	0.3980	0.1545	0.097*	
C4	0.56173 (15)	0.3664 (3)	−0.0467 (3)	0.0850 (10)	
H4A	0.5869	0.3986	−0.0856	0.102*	
C18	0.32429 (16)	0.1780 (4)	0.4223 (3)	0.0940 (12)	
H18A	0.2924	0.2252	0.4062	0.113*	
H18B	0.3553	0.2229	0.4009	0.113*	
C16	0.28390 (18)	−0.0235 (5)	0.5209 (2)	0.1058 (14)	
H16A	0.2547	−0.0710	0.5462	0.127*	
H16B	0.3174	−0.0644	0.5373	0.127*	
C5	0.55830 (15)	0.4125 (4)	0.0386 (3)	0.0916 (11)	
H5A	0.5813	0.4759	0.0574	0.110*	
C17	0.3284 (2)	0.1644 (5)	0.5233 (3)	0.140 (2)	
H17A	0.3613	0.1211	0.5394	0.168*	
H17B	0.3296	0.2461	0.5507	0.168*	
P1	0.15365 (4)	0.14226 (9)	0.20740 (6)	0.0750 (3)	
F1	0.1241 (5)	0.2534 (10)	0.2540 (7)	0.141 (3)	0.65 (2)
F2	0.1364 (4)	0.1889 (13)	0.1147 (8)	0.152 (4)	0.65 (2)
F3	0.2064 (5)	0.2159 (11)	0.2041 (10)	0.136 (4)	0.65 (2)
F4	0.0987 (4)	0.0652 (11)	0.2203 (5)	0.113 (2)	0.65 (2)
F5	0.1803 (6)	0.0238 (11)	0.1705 (8)	0.135 (4)	0.65 (2)
F6	0.1702 (3)	0.0918 (8)	0.3061 (6)	0.108 (2)	0.65 (2)
F1'	0.1419 (7)	0.2894 (14)	0.208 (3)	0.161 (9)	0.35 (2)
F2'	0.1432 (9)	0.147 (2)	0.1016 (11)	0.144 (8)	0.35 (2)
F3'	0.2163 (6)	0.186 (2)	0.1825 (12)	0.106 (5)	0.35 (2)
F4'	0.0981 (6)	0.116 (3)	0.2162 (15)	0.168 (10)	0.35 (2)
F5'	0.1754 (15)	0.0133 (19)	0.192 (2)	0.183 (12)	0.35 (2)
F6'	0.1687 (9)	0.147 (4)	0.3025 (11)	0.185 (10)	0.35 (2)

### Atomic displacement parameters (Å^2^)

**Table:**

	*U*^11^	*U*^22^	*U*^33^	*U*^12^	*U*^13^	*U*^23^
N2	0.0516 (13)	0.0768 (16)	0.0475 (12)	0.0046 (12)	0.0073 (9)	0.0004 (11)
C11	0.0619 (17)	0.0708 (18)	0.0434 (14)	0.0016 (14)	0.0008 (12)	0.0058 (12)
C2	0.0577 (16)	0.0639 (17)	0.0575 (16)	0.0037 (13)	0.0061 (13)	0.0023 (13)
C8	0.0634 (17)	0.0610 (17)	0.0461 (14)	0.0001 (13)	0.0020 (12)	0.0002 (12)
N1	0.0799 (19)	0.094 (2)	0.0675 (17)	−0.0107 (16)	−0.0001 (14)	−0.0165 (15)
C1	0.0620 (17)	0.0611 (17)	0.0566 (16)	−0.0026 (14)	0.0022 (13)	0.0024 (13)
C3	0.072 (2)	0.084 (2)	0.070 (2)	−0.0006 (17)	0.0195 (16)	0.0039 (16)
C15	0.071 (2)	0.085 (2)	0.0637 (18)	−0.0106 (17)	0.0155 (15)	0.0033 (15)
C7	0.0638 (18)	0.076 (2)	0.0481 (15)	0.0040 (16)	0.0055 (13)	−0.0039 (14)
C10	0.087 (2)	0.0641 (17)	0.0537 (16)	0.0120 (16)	0.0176 (15)	0.0115 (13)
C9	0.077 (2)	0.0720 (19)	0.0575 (17)	0.0198 (16)	0.0156 (15)	0.0090 (14)
C14	0.0677 (19)	0.091 (2)	0.0504 (16)	−0.0039 (17)	−0.0038 (13)	0.0087 (15)
C12	0.078 (2)	0.085 (2)	0.101 (3)	0.0250 (19)	0.032 (2)	0.026 (2)
O1	0.123 (2)	0.188 (3)	0.0812 (18)	−0.057 (2)	0.0535 (16)	−0.049 (2)
C13	0.097 (3)	0.0618 (19)	0.106 (3)	0.0170 (18)	0.034 (2)	0.0198 (18)
C6	0.091 (2)	0.083 (2)	0.069 (2)	−0.0196 (19)	−0.0070 (18)	−0.0045 (17)
C4	0.077 (2)	0.085 (2)	0.094 (3)	−0.0058 (19)	0.0180 (19)	0.017 (2)
C18	0.095 (3)	0.093 (3)	0.096 (3)	−0.028 (2)	0.040 (2)	−0.034 (2)
C16	0.103 (3)	0.153 (4)	0.062 (2)	−0.017 (3)	0.027 (2)	0.012 (2)
C5	0.080 (2)	0.088 (3)	0.107 (3)	−0.029 (2)	−0.001 (2)	0.011 (2)
C17	0.140 (4)	0.196 (5)	0.085 (3)	−0.075 (4)	0.052 (3)	−0.065 (3)
P1	0.0695 (6)	0.0775 (6)	0.0778 (6)	0.0104 (4)	−0.0032 (4)	−0.0038 (4)
F1	0.158 (7)	0.082 (4)	0.184 (7)	0.038 (4)	0.042 (4)	−0.015 (4)
F2	0.140 (5)	0.199 (9)	0.116 (7)	0.038 (6)	−0.024 (4)	0.072 (6)
F3	0.095 (5)	0.089 (4)	0.223 (10)	−0.024 (4)	−0.005 (5)	0.038 (5)
F4	0.097 (4)	0.146 (6)	0.096 (4)	−0.045 (4)	−0.004 (3)	−0.003 (3)
F5	0.157 (6)	0.112 (7)	0.138 (5)	0.028 (6)	0.075 (5)	−0.029 (5)
F6	0.095 (4)	0.136 (5)	0.093 (4)	0.011 (3)	−0.012 (2)	0.032 (4)
F1'	0.129 (10)	0.071 (7)	0.28 (3)	0.023 (6)	0.021 (10)	−0.022 (10)
F2'	0.167 (14)	0.198 (15)	0.068 (6)	−0.089 (13)	0.002 (7)	−0.033 (9)
F3'	0.061 (5)	0.143 (13)	0.113 (7)	0.024 (6)	−0.004 (5)	0.031 (7)
F4'	0.059 (6)	0.22 (3)	0.224 (16)	−0.005 (9)	0.043 (7)	−0.003 (13)
F5'	0.22 (2)	0.063 (8)	0.27 (3)	0.026 (9)	0.080 (17)	0.050 (12)
F6'	0.195 (14)	0.30 (3)	0.059 (8)	0.041 (16)	−0.016 (7)	−0.055 (11)

### Geometric parameters (Å, º)

**Table:**

N2—C18	1.482 (4)	O1—C16	1.403 (5)
N2—C15	1.502 (4)	O1—C17	1.423 (5)
N2—C14	1.503 (3)	C13—H13A	0.9300
N2—H2A	0.9100	C6—C5	1.385 (5)
C11—C10	1.362 (4)	C6—H6A	0.9300
C11—C12	1.378 (4)	C4—C5	1.370 (5)
C11—C14	1.513 (4)	C4—H4A	0.9300
C2—C3	1.396 (4)	C18—C17	1.516 (5)
C2—C1	1.409 (4)	C18—H18A	0.9700
C2—C7	1.440 (4)	C18—H18B	0.9700
C8—C9	1.377 (4)	C16—H16A	0.9700
C8—C13	1.384 (4)	C16—H16B	0.9700
C8—C1	1.492 (4)	C5—H5A	0.9300
N1—C7	1.142 (4)	C17—H17A	0.9700
C1—C6	1.386 (4)	C17—H17B	0.9700
C3—C4	1.375 (5)	P1—F4'	1.421 (17)
C3—H3A	0.9300	P1—F6'	1.463 (16)
C15—C16	1.502 (5)	P1—F5'	1.51 (2)
C15—H15A	0.9700	P1—F2	1.527 (8)
C15—H15B	0.9700	P1—F3	1.536 (9)
C10—C9	1.384 (4)	P1—F5	1.543 (9)
C10—H10A	0.9300	P1—F1	1.574 (7)
C9—H9A	0.9300	P1—F2'	1.597 (15)
C14—H14A	0.9700	P1—F1'	1.610 (14)
C14—H14B	0.9700	P1—F4	1.615 (9)
C12—C13	1.384 (5)	P1—F6	1.616 (7)
C12—H12A	0.9300	P1—F3'	1.678 (17)
			
C18—N2—C15	109.6 (2)	C6—C5—H5A	119.8
C18—N2—C14	113.7 (3)	O1—C17—C18	111.0 (3)
C15—N2—C14	110.5 (2)	O1—C17—H17A	109.4
C18—N2—H2A	107.6	C18—C17—H17A	109.4
C15—N2—H2A	107.6	O1—C17—H17B	109.4
C14—N2—H2A	107.6	C18—C17—H17B	109.4
C10—C11—C12	118.7 (3)	H17A—C17—H17B	108.0
C10—C11—C14	120.4 (3)	F4'—P1—F6'	98.6 (13)
C12—C11—C14	120.8 (3)	F4'—P1—F5'	100.7 (18)
C3—C2—C1	121.4 (3)	F6'—P1—F5'	95.1 (17)
C3—C2—C7	119.0 (3)	F4'—P1—F2	83.7 (11)
C1—C2—C7	119.5 (3)	F6'—P1—F2	158.7 (14)
C9—C8—C13	117.6 (3)	F5'—P1—F2	105.3 (13)
C9—C8—C1	121.2 (3)	F4'—P1—F3	160.1 (12)
C13—C8—C1	121.1 (3)	F6'—P1—F3	79.0 (11)
C6—C1—C2	116.7 (3)	F5'—P1—F3	99.2 (14)
C6—C1—C8	123.3 (3)	F2—P1—F3	91.6 (6)
C2—C1—C8	120.0 (3)	F4'—P1—F5	107.3 (14)
C4—C3—C2	119.5 (3)	F6'—P1—F5	105.6 (15)
C4—C3—H3A	120.2	F2—P1—F5	93.7 (7)
C2—C3—H3A	120.2	F3—P1—F5	92.3 (8)
C16—C15—N2	110.7 (3)	F4'—P1—F1	69.1 (11)
C16—C15—H15A	109.5	F6'—P1—F1	70.1 (14)
N2—C15—H15A	109.5	F5'—P1—F1	159.6 (10)
C16—C15—H15B	109.5	F2—P1—F1	91.4 (6)
N2—C15—H15B	109.5	F3—P1—F1	91.8 (5)
H15A—C15—H15B	108.1	F5—P1—F1	173.4 (5)
N1—C7—C2	178.5 (3)	F4'—P1—F2'	87.5 (11)
C11—C10—C9	120.9 (3)	F6'—P1—F2'	173.3 (14)
C11—C10—H10A	119.6	F5'—P1—F2'	86.4 (14)
C9—C10—H10A	119.6	F3—P1—F2'	94.3 (8)
C8—C9—C10	121.2 (3)	F5—P1—F2'	74.8 (10)
C8—C9—H9A	119.4	F1—P1—F2'	110.0 (9)
C10—C9—H9A	119.4	F4'—P1—F1'	90.9 (12)
N2—C14—C11	113.5 (2)	F6'—P1—F1'	90.5 (12)
N2—C14—H14A	108.9	F5'—P1—F1'	166.2 (12)
C11—C14—H14A	108.9	F2—P1—F1'	68.3 (10)
N2—C14—H14B	108.9	F3—P1—F1'	69.4 (7)
C11—C14—H14B	108.9	F5—P1—F1'	153.1 (13)
H14A—C14—H14B	107.7	F2'—P1—F1'	86.7 (11)
C11—C12—C13	120.6 (3)	F6'—P1—F4	96.0 (13)
C11—C12—H12A	119.7	F5'—P1—F4	81.5 (13)
C13—C12—H12A	119.7	F2—P1—F4	93.0 (6)
C16—O1—C17	109.3 (3)	F3—P1—F4	175.0 (6)
C8—C13—C12	120.9 (3)	F5—P1—F4	89.4 (7)
C8—C13—H13A	119.5	F1—P1—F4	86.1 (6)
C12—C13—H13A	119.5	F2'—P1—F4	90.7 (7)
C5—C6—C1	121.8 (3)	F1'—P1—F4	110.5 (8)
C5—C6—H6A	119.1	F4'—P1—F6	94.7 (11)
C1—C6—H6A	119.1	F5'—P1—F6	74.9 (13)
C5—C4—C3	120.1 (3)	F2—P1—F6	178.3 (5)
C5—C4—H4A	119.9	F3—P1—F6	90.0 (5)
C3—C4—H4A	119.9	F5—P1—F6	86.8 (6)
N2—C18—C17	110.0 (3)	F1—P1—F6	88.0 (4)
N2—C18—H18A	109.7	F2'—P1—F6	161.3 (9)
C17—C18—H18A	109.7	F1'—P1—F6	111.8 (12)
N2—C18—H18B	109.7	F4—P1—F6	85.4 (4)
C17—C18—H18B	109.7	F4'—P1—F3'	170.8 (13)
H18A—C18—H18B	108.2	F6'—P1—F3'	89.0 (11)
O1—C16—C15	111.3 (3)	F5'—P1—F3'	83.6 (14)
O1—C16—H16A	109.4	F2—P1—F3'	87.4 (7)
C15—C16—H16A	109.4	F5—P1—F3'	75.2 (9)
O1—C16—H16B	109.4	F1—P1—F3'	109.2 (8)
C15—C16—H16B	109.4	F2'—P1—F3'	84.6 (9)
H16A—C16—H16B	108.0	F1'—P1—F3'	83.9 (9)
C4—C5—C6	120.4 (3)	F4—P1—F3'	164.7 (8)
C4—C5—H5A	119.8	F6—P1—F3'	94.3 (6)

### Hydrogen-bond geometry (Å, º)

**Table:**

*D*—H···*A*	*D*—H	H···*A*	*D*···*A*	*D*—H···*A*
N2—H2*A*···N1^i^	0.91	2.04	2.942 (4)	171
C10—H10*A*···F1^ii^	0.93	2.43	3.296 (9)	155
C14—H14*A*···F3	0.97	2.39	3.355 (12)	171
C15—H15*A*···F6	0.97	2.46	3.377 (8)	158
C15—H15*B*···F3^ii^	0.97	2.48	3.412 (10)	161
C15—H15*B*···F3′^ii^	0.97	2.54	3.51 (2)	172
C10—H10*A*···F1′^ii^	0.93	2.45	3.26 (2)	145
C14—H14*B*···F1′^ii^	0.97	2.38	3.097 (16)	130
C5—H5*A*···F2′^iii^	0.93	2.48	3.41 (2)	178
C17—H17*B*···F6′^iv^	0.97	2.47	3.29 (3)	143

Symmetry codes: (i) *x*, −*y*, *z*+1/2; (ii) −*x*+1/2, *y*−1/2, −*z*+1/2; (iii) *x*+1/2, *y*+1/2, *z*; (iv) −*x*+1/2, −*y*+1/2, −*z*+1.
